# Heat-mortality relationship in North Carolina: Comparison using different exposure methods

**DOI:** 10.1038/s41370-023-00544-y

**Published:** 2023-04-07

**Authors:** Hayon Michelle Choi, Michelle L. Bell

**Affiliations:** School of the Environment, Yale University, New Haven, CT USA

**Keywords:** Health Studies, Exposure Modeling, Personal Exposure.

## Abstract

**Background:**

Many studies have explored the heat-mortality relationship; however, comparability of results is hindered by the studies’ use of different exposure methods.

**Objective:**

This study evaluated different methods for estimating exposure to temperature using individual-level data and examined the impacts on the heat-mortality relationship.

**Methods:**

We calculated different temperature exposures for each individual death by using a modeled, gridded temperature dataset and a monitoring station dataset in North Carolina for 2000–2016. We considered individual-level vs. county-level averages and measured vs. modeled temperature data. A case-crossover analysis was conducted to examine the heat-mortality risk under different exposure methods.

**Results:**

The minimum mortality temperature (MMT) (i.e., the temperature with the lowest mortality rate) for the monitoring station dataset was 23.87 °C and 22.67 °C (individual monitor and county average, respectively), whereas for the modeled temperature dataset the MMT was 19.46 °C and 19.61 °C (individual and county, respectively). We found higher heat-mortality risk while using temperature exposure estimated from monitoring stations compared to risk based on exposure using the modeled temperature dataset. Individual-aggregated monitoring station temperature exposure resulted in higher heat mortality risk (odds ratio (95% CI): 2.24 (95% CI: 2.21, 2.27)) for a relative temperature change comparing the 99th and 90th temperature percentiles, while modeled temperature exposure resulted in lower odds ratio of 1.27 (95% CI: 1.25, 1.29).

**Significance:**

Our findings indicate that using different temperature exposure methods can result in different temperature-mortality risk. The impact of using various exposure methods should be considered in planning health policies related to high temperatures, including under climate change.

**Impact Statement:**

1. We estimated the heat-mortality association using different methods to estimate exposure to temperature.
2. The mean temperature value among different exposure methods were similar although lower for the modeled data, however, use of the monitoring station temperature dataset resulted in higher heat-mortality risk than the modeled temperature dataset.
3. Differences in mortality risk from heat by urbanicity varies depending on the method used to estimate temperature exposure.

## Introduction

A substantial number of studies on the impact of temperature on mortality provide evidence for an increased risk of death from extreme temperature (e.g., low and high ambient temperature) [1–3]. Previous studies often investigated heat-related mortality using daily values of temperature observed at a single weather station and aggregating the exposure into county- or city-level averages. While the use of monitoring stations to estimate exposure has the strength of actual measurements, this approach could pose limitations in terms of study area and population. Monitoring stations are often located in or near cities, which are often hotter than surrounding rural areas due to the urban heat island effect. This could result in the inability to study other, more rural populations or in biased or inaccurate estimated effects for rural populations or others living far away from the monitoring stations [4].

Many recently, modeled weather datasets at a global and local levels are used widely to estimate exposure to temperature and to explore the climate change impacts [5]. These gridded modeled exposure datasets incorporated mixed methods and spatial interpolation to accurately derive temperature estimates at a higher resolution [6, 7]. For instance, global satellite imagery is publicly available, and are now commonly used in scientific research. Even though the modeled weather dataset can be highly useful for research, especially for estimating exposure for locations without monitors, there are limitations. Such datasets cannot be fully validated at locations without monitors, meaning the models have more certainty in some types of areas than others. Also, modeled temperature datasets could have measurement error in places with different elevation level or certain conditions, such as clouds [8].

Some studies have applied temperature datasets from gridded model or monitoring stations to individual-level health data [9, 10]. However, most studies use one form of exposure data and do not compare the exposure values among different exposure methods or the resulting health effect estimates. Few studies have estimated the effect of different temperature exposure methods on estimated risk of mortality [4, 11, 12]. The limited research has inconsistent results. One study compared temperature-related mortality using temperature measurements from a single weather station and modeled meteorological dataset for 113 cities in US [11]. The results showed no significant difference in temperature-related mortality between these two methods. Another study showed higher temperature-mortality associations using exposure data with more spatial variability compared to temperatures based on monitors [4]. Earlier research lacks fine-scale spatial temperature data [13, 14] or neglects county or region-specific mortality [15]. Research is needed on how different approaches to estimate exposure to temperature impact the resulting health estimates. This is particularly important given the anticipated increase in temperature-related health risk under a changing climate [16, 17]. Therefore, we compared temperature exposure based on multiple exposure methods and explored the effect of various exposure methods on estimated temperature-mortality associations.

In this study, we evaluated how temperature-mortality relationships vary by use of method to estimate exposure. We consider exposure based on measurements (i.e., weather station monitors) and estimates (gridded modeled temperature data), and two types of health data (individual-level data compared aggregated spatial data). Specifically, we estimated the association between mean temperature and mortality using: 1) individual-level temperature estimates constructed from modeled data, 2) county-aggregated temperature from modeled data, 3) individual-level temperature estimates from monitoring stations, and 4) county-aggregated temperature estimated from monitoring stations.

## Material and Methods

### Study site

North Carolina (NC), a state in the southeastern region of the United Sates, was examined for this study. NC’s 100 counties are mostly in the humid subtropical climate zone, experiencing hot and humid summers. However, western NC lies in the subtropical highland climate, with mountainous areas that experience cool summers. Three different eco-regions (Mountains, Piedmont, and Coastal Plain) exist within NC, as do both urban and rural areas [18]. The diversity of NC in terms of regional characteristics facilitates study of the possible difference in environmental exposure that might relate to heat related mortality risks.

### Data

#### Mortality dataset

We obtained individual-level mortality data for NC for May to September for the years 2000 to 2016 from the NC State Center for Health Statistics, Vital Statistics Department. For each participant, mortality data included date of death, residential county, coordinate of residence, and demographic variables (e.g., sex, age, and race/ethnicity). We classified mortality data as total mortality as all causes of death except external causes (International Classification of Diseases, ICD-10, A00-R99). We excluded participants with incomplete data for any variable.

#### Meteorological datasets

We used two types of temperature datasets, a modeled dataset and measurements from monitoring stations, and considered two different methods of spatial scale: individual-level exposure based on the participant’s geolocated residence and county aggregated exposure based on the county of residence. These approaches are commonly used in epidemiological research on temperature. The modeled temperature data are from Parameter-elevation Regressions on Independent Slopes Model (PRISM), a publicly available gridded dataset, which spatially interpolated weather observations from various observation networks using multivariate regression models that adjust for elevation, topography, and other geophysical characteristics [19]. The PRISM data are reported on a daily basis and at high spatial resolution (4 × 4 km grid), and we used the PRISM dataset for 2000 to 2016 [7]. The algorithms and further details are described elsewhere [20, 21]. A previous study showed good agreement between measured and gridded weather data [22]. Monitoring data were obtained from the NC State Climate Office for 2000 to 2016 during May to September.

#### Exposure methods

We considered four exposure methods based on combinations of modeled versus measured temperature and individual- versus county-level exposure.

A) *Modeled temperature data* matched to the *individual residence*: We used estimated gridded weather data to estimate exposure for each individual within a 5 km buffer of their residence. Area-weighted averaging was used to convert the gridded dataset into estimates within buffers. The area-weighted average was computed based on the different area of grid cells within a 5 km buffer. For instance, if the 5 km buffer is 60% in PRISM grid cell A and 40% in grid cell B, we would compute the temperature of that 5 km buffer as an area-weighted average of the temperatures of grid cells A and B (i.e., 0.6 × Temperature_CellA_ + 0.4 × Temperature_CellB_).

B) *Modeled temperature data* matched to the *residential county*: County-level values were calculated based on the average of gridded values with locations in a given county using the area-weighted average.

C) *Monitoring station temperature data* matched to the *individual residence*: We derived daily temperature exposure estimates based on individual residences and monitoring stations by applying a 5 km buffer around each residence and assigning the average temperature of the monitors within that buffer. If one individual was within a 5 km buffer of multiple monitors, we averaged the monitor values, and one individual was excluded from the analysis if one had no monitor station within 5 km of residence.

D) *Monitoring station temperature data* matched to the *residential county*: County-level temperature values were calculated as the area-weighted average values from monitors in the county or within 5 km of the county edge. If no monitor was present within a county or within 5 km of its boundary, the county was not included in this analysis.

For both methods based on monitors, only individuals within 5 km of at least one monitor were included in analysis of this exposure method. We excluded monitoring stations that were newly added and/or removed during the study period with a time span of less than 13 years, which excluded 25 stations.

We calculated the correlation between pairs of monitoring stations and the correlation between the different exposure methods.

#### Urbanization and regional dataset

Data on the designations of urbancity were obtained from the Census Bureau, which classified urbanization into three types: urbanized areas, urban cluster, and rural at the county level. Urbanized areas are areas with 50,000 or more people, urban cluster are areas with at least 2500 but fewer than 50,000 people, and rural areas have less than 2500 people [23]. The three principal regions of North Carolina are the Mountain, Piedmont, and Coastal Plain (from west to east) [24].

### Statistical Analysis

A conditional logistic regression model was used in a case-crossover study design to estimate the effect of heat exposure on mortality among different exposure methods. For each individual, we identified the date of death as a ‘case’ and proximate days as ‘controls’ within a 28-day window. Case days were matched on day-of-week (DOW) within a 28-day window of the case day to identify controls for each case. For each record, a distributed temperature lag of up to one day after the day of death (Lag 0–1) was compared to corresponding temperatures on the control days in order to calculate an odds ratio (OR) (95%CI) (a comparison of risk between days with case and control temperatures). Lag 0–1 was selected based on previous heat-mortality studies on the different lag periods affecting mortality risks, finding that heat-related mortality occurred in a short-term (Lag 0–1) lag period [25, 26]. Odds ratio comparing the heat-mortality risk of relative temperature changes (99th temperature percentile and 90th temperature percentile was calculated separately for the four exposure methods) and absolute temperature changes (30.0 °C and 27.6 °C) were analyzed. The absolute temperature values were calculated based on the 99th temperature percentile and 90^th^ temperature percentile based on the average NC temperature. The summary statistics for each temperature exposure method are shown in Table S1. Also, the heat-mortality odds ratio for different categories of urbanicity (Urban Area, Urban Cluster, and Rural) were examined through separate subgroup analysis.

R software (version 4.0.3) and SAS were used to conduct the analysis. Results were considered as statistical significance if it fulfilled two tailed *p* < 0.05.

## Results

### Descriptive results

The general characteristics of the subpopulations included in mortality analysis under different methods to estimate exposure to temperature are presented in Table 1 and summary statistics for temperature values for mortalities (Lag 0) by subpopulation are shown in Table 2. There were 351,907 deaths registered in North Carolina during the study period. Most were located in the Piedmont counties (177,250, 50.4%), from urban cluster counties (200,363, 56.9%), non-Hispanic White (265,866, 75.6%), and over 65 years (231,388, 65.8%). The number of persons included in exposure estimates based on monitoring data within specified buffers (i.e., people living with 5 km of a monitor) was 109,569, and the number of persons included in exposure estimates based on county-aggregated data from monitor stations (i.e., people living in a county with a monitor) was 118,848. These individuals’ characteristics were similar to the total dataset based on all mortalities, with non-Hispanic White (Individual; County: 78,642, 71.8%; 87,776, 73.9%), and mostly over 65 years (Individual; County: 67,388, 61.5%; 74,501, 62.7%).

**Table 1:** Descriptive statistics for populations for mortality data included under different exposure methods (May–September, 2000–2016).

		PRISM	Monitoring station	
			Individual residence	Residential county
		*N* (%)	*N* (%)	*N* (%)
	Total	351,907	109,569	118,848
Region	Piedmont	177,250 (50.4%)	42,625 (38.9%)	53,311 (44.9%)
	Mountain	61,228 (17.4%)	22,142 (20.2%)	22,978 (19.3%)
	Coastal	113,429 (32.2%)	44,802 (40.9%)	42,559 (35.8%)
Urbanicity	Urban Area	18,201 (5.2%)	5,236 (4.8%)	5,405 (4.6%)
	Urban Cluster	200,363 (56.9%)	70,121 (64.0%)	79,261 (66.7%)
	Rural	133,343 (37.9%)	34,212 (31.2%)	34,182 (28.8%)
Race	Non-Hispanic White	265,866 (75.6%)	78,642 (71.8%)	87,776 (73.9%)
	Non-Hispanic Black	76,261 (21.7%)	28,619 (26.1%)	28,577 (24.0%)
	Hispanic	4,474 (1.3%)	1,216 (1.1%)	1,379 (1.2%)
	Non-Hispanic Asian/Hawaiian Pacific Islander	1,910 (0.5%)	632 (0.6%)	665 (0.6%)
	Non-Hispanic Other	3,396 (1.0%)	460 (0.4%)	451 (0.4%)
Sex	Female	171,141 (48.6%)	55,549 (50.7%)	59,848 (50.4%)
	Male	180,754 (51.4%)	54,019 (49.3%)	58.999 (49.7%)
Age	Mean (sd)	67.7 (21.2)	63.9 (26.0)	64.9 (25.2)
	< 65	120,519 (34.3%)	42,181 (38.5%)	44,347 (37.3%)
	≥ 65	231,388 (65.8%)	67,388 (61.5%)	74,501 (62.7%)

*sd* standard deviation

**Table 2:** Descriptive statistics for temperature exposures for mortalities (Lag 0) for different exposure methods (May–September 2000–2016).

		PRISM (°C)		Monitoring station (°C)	
		Individual	County	Individual	County
		Mean (SD)	Mean (SD)	Mean (SD)	Mean (SD)
	Total	23.4 (3.8)	23.4 (3.9)	23.6 (4.2)	23.6 (4.1)
Region	Piedmont	23.5 (3.6)	23.5 (3.7)	23.4 (3.6)	23.4 (3.6)
	Mountain	20.7 (3.5)	20.5 (3.7)	20.0 (3.3)	20.2 (3.2)
	Coastal	24.7 (3.5)	24.7 (3.7)	25.4 (3.9)	25.6 (3.8)
Urbanicity	Urban Area	24.3 (3.5)	24.4 (3.6)	24.5 (3.4)	24.4 (3.4)
	Urban Cluster	23.6 (3.9)	23.6 (3.8)	24.1 (4.2)	23.9 (4.1)
	Rural	22.9 (3.9)	22.9 (4.0)	22.3 (4.0)	22.5 (3.9)
Race	Non-Hispanic White	23.1 (3.8)	23.1 (3.9)	22.8 (3.9)	23.0 (3.8)
	Non-Hispanic Black	24.4 (3.7)	24.4 (3.9)	25.5 (4.3)	25.4 (4.4)
	Hispanic	23.7 (3.5)	23.7 (3.6)	23.6 (3.3)	23.2 (3.3)
	Non-Hispanic Asian/Hawaiian Pacific Islander	23.7 (3.7)	23.7 (3.8)	23.8 (3.6)	23.6 (3.6)
	Non-Hispanic Other	24.3 (3.6)	24.3 (3.7)	24.2 (3.7)	24.0 (3.6)
Sex	Female	23.5 (4.0)	23.5 (4.0)	23.9 (4.4)	23.8 (4.3)
	Male	23.3 (3.9)	23.3 (3.9)	23.2 (4.0)	23.3 (3.8)
Age	< 65	23.8 (3.9)	23.7 (4.1)	24.5 (4.6)	24.5 (4.7)
	≥ 65	23.2 (3.8)	23.2 (3.9)	23.0 (3.8)	23.0 (3.7)

*SD* Standard deviation; The number of participants varied by exposure method (See Table 1).

The mean temperature among different exposure methods were fairly consistent, ranging from 23.4 °C to 23.6 °C. The Coastal region, urban areas, Non-Hispanic Black persons, females, and people under 65 years had higher temperature values for all four exposure methods. This was similar when comparing the exposure estimates within the subset of individuals with estimates under all the different exposure methods (Table S2). Figure S1 shows the locations of the 25 monitoring stations used in this study and the three regions (Mountain, Piedmont, Coastal Plain). The correlation between each pair of monitoring stations compared to the distance between pairs of monitor stations is shown in Fig. S2.

**Fig. 1:**
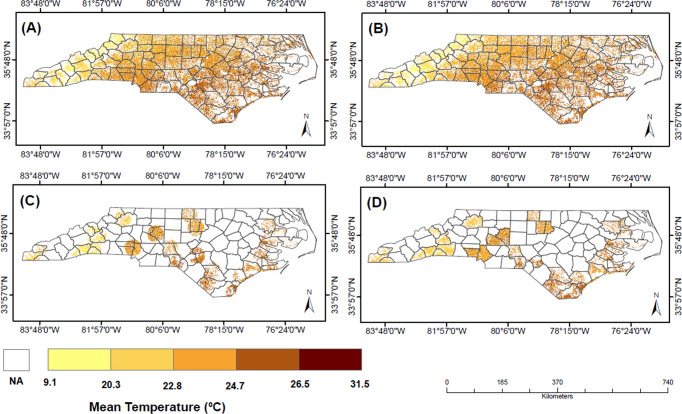
The mean temperature map for each individual death based on different exposure methods. Exposures based on. **A** Modeled temperature data matched to the individual residence, (**B**) modeled temperature data matched to the residential county, (**C**) monitoring temperature data matched to the individual residence, and (**D**) monitoring temperature data matched to the residential county. Each dot represents an individual in the mortality dataset. The colors reflect temperature exposure estimates for those individuals one of four exposure methods. White areas reflect locations where either no estimate could be made for that exposure method or no participant resided.

The mean temperature distribution for each exposure method is shown in Fig. 1. Exposures based on modeled temperature dataset (PRISM) is very similar between individual- and county-aggregated temperature (Fig. 1A, B). For exposures based on monitoring data (Fig. 1C, D), there are some counties that are not assigned a mean temperature value using this method due to the location of the monitoring stations. The overall temperature distribution was generally similar between exposure methods, although levels were slightly higher for monitoring stations compared to the modeled temperature dataset. The temperatures were lower in the West (Mountain region) and higher in the East-Southern areas (Coastal and Piedmont region). Overall, the correlation among different exposure methods were high. The correlation between exposures based on modeled temperature matched to the residential county (B) and exposures based on monitoring data matched to the residential county (D) was 0.9, as was the correlation between (B) and exposures based on monitoring data matched to individual residence (C). The correlation between exposures based on modeled data matched to individual residence (A) and (D) was 0.85 and the correlation for and (A) and (C) was 0.86.

**Fig. 2:**
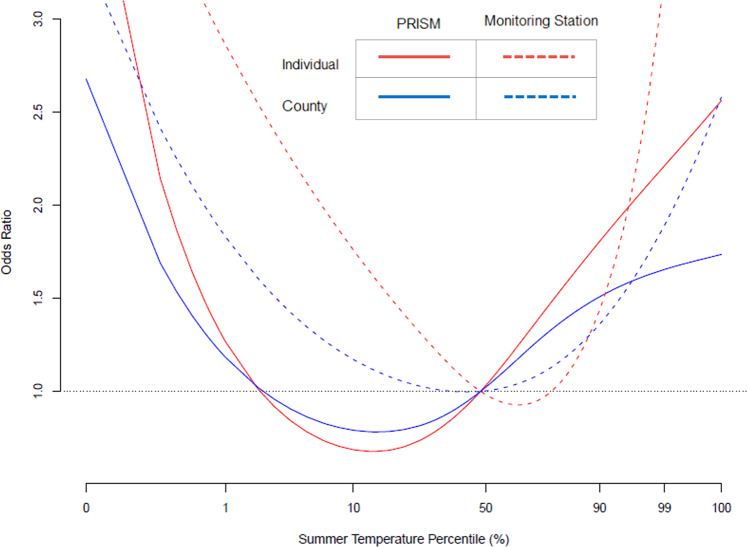
The exposure-response curve for each exposure method using a subset of individuals in all exposure methods (*n* = 109,569). Solid lines indicate results based on the gridded, modeled temperature dataset (PRISM) and dashed lines reflect results based on monitoring station temperature. Blue lines indicate results based on exposure matched to the individual residence, and red lines indicate results based on exposure matched to the residential county aggregated. The odds ratio was centered (OR = 1) for the overall temperature mean value of 23.2 °C.

Figure 2 shows the pooled cumulative exposure-response relationship between temperature and mortality for different exposure methods. We observed a U-shaped curve for all four methods. We found higher risks using exposure based on monitoring stations compared to the modeled PRISM dataset. Also, the minimum mortality temperature (MMT), or optimal temperature, was higher using exposure methods based on monitoring station data compared to the modeled temperature data. The MMT for the monitoring station dataset was 23.87 °C and 22.67 °C (individual and county, respectively), whereas for the modeled temperature dataset the MMT was 19.46 °C and 19.61 °C (individual and county, respectively).

Overall, the heat mortality risk was higher when using the monitoring station dataset compared to the modeled temperature dataset, although results were not statistically different (Table 3). When assessing exposure based on monitoring data, the heat mortality risk was higher using estimates linked to individual residence (OR 2.24, 95% CI: 2.21, 2.27, comparing the relative temperature changes (99^th^ temperature percentile and 90^th^ temperature percentile) than estimates linked to residential county (OR 1.27, 95% CI: 1.25, 1.29). Estimates based on individual residence were also higher than those based on residential county using the modeled PRISM exposure dataset (OR 1.16, 95% CI: 1.14, 1.17 vs. OR 1.08, 95% CI: 1.06, 1.10).

**Table 3:** Heat related mortality (OR, 95% CI) estimated from conditional logistic regression models used within a case-crossover framework for different exposure methods using the subset of individuals included in all exposure methods (*n* = 109,569).

	Odds Ratio (95% CI)			
	Temperature based on individual residence		Temperature based on residential county	
	PRISM	Monitoring stations	PRISM	Monitoring stations
Relative	1.16 (1.14, 1.17)	2.24 (2.21, 2.27)	1.08 (1.06, 1.10)	1.27 (1.25, 1.29)
Absolute	1.25 (1.22, 1.28)	2.23 (2.20, 2.27)	1.12 (1.08, 1.14)	1.39 (1.36, 1.42)

Odds ratio comparing the heat-mortality risk of relative temperature changes (99th temperature percentile and 90th temperature percentile) and absolute temperature changes (30.0 °C and 27.6 °C).

The results shown in Fig. 2 and Table 3 are based on the subset of individuals that have exposure estimates under all four methods (*n* = 109,569). This includes participants who lived far from monitors. We also conducted analysis using all the individuals with data available for each exposure method, resulting in different numbers of participants by exposure method (109,569 to 351,907) (Table S3 and Fig. S3). For each of the four exposure methods, the odds ratios were similar for analysis using the subset of individuals with exposure estimates for all exposure methods and the analysis using all participants. Subsequent analysis is based on all participants with exposure data available (Table S3 and Fig. S3).

The subgroup analysis estimating the heat-mortality odds ratio by different levels of urbanicity is shown in Table 4. When using the modeled temperature dataset to estimate exposure, urban areas had higher heat-mortality odds ratio than urban cluster or rural regions (OR for urban areas at 1.31, 95% CI: 1.25, 1.38, and 1.23, 95% CI: 1.16, 1.29 based on exposure matched to individual residence and residential county, respectively). Similarly, the rural regions showed the highest heat-mortality odds ratio compared to the urban area and urban cluster when using the monitoring stations to assess exposure (OR 1.36, 95% CI: 1.26, 1.41, and OR 1.37, 95% CI: 1.29, 1.41; individual and county, respectively).

**Table 4:** Heat related mortality (OR, 95% CI) estimated from conditional logistic regression models used within a case-crossover framework by levels of urbanicity for different exposure methods using the subset of individuals included in all exposure methods (n = 109,569).

	Odds Ratio (95% CI)			
	Temperature based on individual resident		Temperature based on residential county	
	PRISM	Monitoring stations	PRISM	Monitoring stations
Urban Area	1.11 (1.02, 1.21)	1.11 (1.02, 1.21)	1.15 (1.05, 1.26)	1.06 (1.00, 1.12)
Urban Cluster	1.22 (1.12, 1.33)	1.24 (1.14, 1.27)	0.96 (0.87, 0.97)	1.35 (1.27, 1.38)
Rural	1.41 (1.29, 1.45)	1.36 (1.26, 1.41)	1.24 (1.13, 1.28)	1.37 (1.29, 1.41)

Odds ratio comparing the heat-mortality risk of relative temperature changes (99th temperature percentile and 90th temperature percentile).

The heat-mortality risk changed when using all individuals (i.e., under all exposure methods) compared to using the subset of individuals with data for all exposure methods (Table S4). Compared to the main findings based on subset of individuals (Table 4), results based on modeled temperature data were lowest when using individual residence for exposure for urban cluster compared to other region (urban area and rural) and highest for rural area, whereas when using residential county results were low for urban cluster and rural areas compared to the urban area. Using data for all individuals with exposure data under all approaches, the urban area was estimated to have the highest heat-mortality risk compared to other urban cluster or rural region (OR (95% CI): 1.31 (1.25, 1.38) and 1.23 (1.16, 1.29) for urban areas using modeled temperature data for individual residence and residential county, respectively).

## Discussion

In this study, we found significant estimated effects of heat on mortality when using different exposure methods. The heat related mortality odds ratio was highest when using the monitoring station dataset among the four exposure methods linked to individual residence, and highest when using the modeled PRISM dataset among the four exposure methods linked to residential county. Also, the heat-mortality risk was different by level of urbancity. Rural region had the higher heat-mortality risk than urban areas or urban cluster when using either the modeled, gridded exposure or the monitoring station dataset. When using the modeled temperature dataset, the heat-mortality risk was lower using county-aggregated exposure compared to individual level for urban cluster and rural region. However, the heat-related mortality risk was higher in urban cluster region using the individual-level exposure data compared to the county-aggregated monitoring station exposure dataset. These study results indicate that using a different method to estimate exposure, such as a different dataset and different level of aggregation, while producing similar overall results, could result in different estimates of the relative health burden related to temperature.

Even though the mean temperature value among the four exposure methods were similar, the heat-related mortality risk was different. Exposure based at the individual residence resulted in a higher heat-mortality risk compared to use of exposure based on residential county using either the monitoring station temperature datasets or modeled exposure dataset. Overall, the high-resolution modeled, gridded temperature dataset resulted in lower heat-mortality risk compared to estimates based on the monitoring station temperature dataset. To date, most studies used weather station datasets when assessing temperature-related health effects. Since the monitoring stations are usually placed near populated areas, they may be close to the true average temperature exposure of the study population in ecological studies [4], however, they may not provide as good representation of temperatures in rural regions. In studies using modeled exposure, populations far from monitors are either excluded or have more uncertain exposure estimates. These populations can have different population characteristics as well (e.g., race/ethnicity, urbanicity). Our study results found different health effects when using different exposure methods, indicating that the choice of exposure method matters. Although all methods indicate increased risk of mortality under heat, the level of that risk differed by exposure method. This can have important implications for decision-makers, such as the development of heat action plans, and estimates of the health impacts of temperature. Further, estimates of the health consequences of climate change would be affected by the different risk estimates.

In our study, we found different heat-mortality risk by level of urbanicity. Importantly, whether rural or urban areas have the highest heat-mortality risk depended on the type of exposure method. This suggests that use of exposure method can also influence results in studies of environmental justice and other research on vulnerability or susceptibility. When examining the heat-mortality risk using all individuals, rural areas had the highest heat-mortality risk under most exposure approaches, but urban areas had higher heat-mortality risks than rural areas when using modeled temperature data based on residential county. This could be explained by the different individual characteristics included in the populations under different subgroup analysis. The people included in the monitoring station exposure analysis had a higher percentage of urban cluster regions and lower percentage of urban area and rural regions compared to the gridded modeled exposure analysis. This is due to the location of the monitoring stations [4], which are mostly located in more urban settings. Therefore, it is important for future studies to consider where the study site is located and what type of temperature exposure methods is being used. Often the type of exposure method selected is based on the availability of the underlying health data (e.g., aggregated health data vs. geocoded location of study participants). If the study area is urbanized, there may be sufficient monitoring stations to estimate temperature-related exposures from measurements, however, studies in rural regions may have a sparser monitoring network that might lead to exposure misclassification. In this case, modeled gridded temperature datasets may be considered, although this brings different limitations as the exposure data would be modeled and exposure uncertainty may differ by participant. The population included in the analysis based on monitoring data was more urban and had a higher percentage of non-Hispanic Black participants than the population based on the modeled temperature data. Also, when using the subset of individuals with exposure estimates under all four methods, results were different regarding risk across levels of urbancity. This could indicate that when estimating the temperature-related health burden in rural regions, using the monitoring station dataset can yield different health results. Most temperature-mortality studies have focused on cities, and most studies including rural regions relied on weather monitoring stations, which are often sparse in rural areas. Thus, the populations studied, and the estimated variation in risks across populations, could differ.

Some studies showed different results from our study. Guo et al. [12] estimated the association between temperature and mortality in Brisbane, Australia using three exposure methods: a time series temperature exposure from a single weather station, a time series averaging across three weather stations, and spatially interpolated (via kriging) temperature estimates for each administrative areas within Brisbane. These three exposures yielded similar effect sizes for both hot and cold temperatures. A study conducted in Paris found little difference in temperature-mortality relationships among different exposure definitions (single monitoring station and population-weighted daily estimates from multiple monitoring stations) [27]. However, while these studies compared results across different exposure methods, they considered only methods based on monitoring data. They also focused on a single city, whereas we considered both rural and urban areas.

Several strengths of this study are that, to the best of our knowledge, it is the first study to compare heat-mortality effect estimates based on different exposure approaches consider both the individual and county aggregated temperature based on both a modeled, gridded dataset and monitoring station dataset. We used the case-crossover study design, which inherently adjusts for all time-invariant confounders. This study included rural populations that are often excluded from epidemiological research based on monitoring networks and assessed effect modification by urbanicity. However, further studies are needed, such as exploring various potential effect modification on heat-mortality relationship when using different exposure methods, such as by access to greenspace. Also, our study results may not be generalizable to different study areas, as the populations, monitoring networks, and accuracy of modeled data may differ.

There are some limitations to this study. We did not account for how the relationship between heat and mortality could differ by air conditioning, including central air conditioning and window units. Nearly 90% of the US household have access to any air conditioning equipment (i.e., central air conditioning or window units) [28], and this rate is similar in North Carolina, where 84% of households have a central air conditioning system [28]. Data are limited on air conditioning, especially for the use of air conditioning, as opposed to its prevalence, as well as window units versus central air conditioning, and other forms of cooling such as electric or hand fans. Having access to air conditioning system does not mean each individual is actually using the air conditioning system. According to the 2015 US Census, 35% or more of households in North Carolina experience energy poverty. Individuals experiencing energy poverty may not be able to pay for the energy needed to use an air conditioning system, even if such a system is installed [29]. Studies investigating the role of air conditioning on heat-mortality relationship are largely based on air conditioning data on average across a country or at the city level [25, 30, 31]. These studies also generally used data on prevalence rather than use of air conditioning. Existing temperature-mortality studies have noted limitations in accurately reflecting the individuals’ exposed indoor temperature, even when considering air conditioning [32–34]. More research and data are needed on how air conditioning influences the heat-mortality relationship including types of units (e.g., central, window units), the use of air conditioning versus its prevalence, and a multi-city scale including urban and rural areas.

Another limitation of this study is in the use of ambient temperature for exposure. This does not account for differences across populations due to indoor/outdoor activity patterns and the corresponding temperatures, which would affect the human health. Some studies reported the relationship between indoor temperature and health, with findings consistent with our study results. High indoor temperature was associated with poor self-rated health in England [35]. Also, a study in Texas found indoor heat was associated with adverse health effects, especially mortality [36]. Many studies have investigated the correlation between indoor and outdoor temperature, and the results vary depending on the region and households. A study in Germany showed poor correlation between outdoor and indoor temperature [37], whereas, a study in Boston found a strong correlation of 0.91 at warm outdoor temperatures [38]. Also, a study conducted for Seoul, Korea showed that outdoor temperature and apparent temperature are sufficient indicators for indoor conditions [35]. Indoor conditions vary between households, but correspond to outdoor conditions [39, 40]. Although the ambient temperature is particularly relevant for policy, as decision-makers often develop policies for ambient levels (e.g., heat warning systems), further work is needed to assess how the heat-mortality relationship differs by subpopulation in relation to indoor temperatures and indoor/outdoor activity patterns, which can relate to different personal exposures.

There are several topics that warrant future research. In this study we analyzed the outdoor temperature as the exposure, although other studies explored a range of metrics including the heat index, which considers the temperature and humidity. Many studies found association between heat index and mortality [41–43]. However, the heat index used in previous studies were generated from different methods and algorithms, which could result in inconsistent results [44]. Future studies could consider more complex aspects of exposure to high temperatures such as the heat index, indoor temperatures, and indoor/outdoor activity patterns, including how different methods of assessing exposure impact estimated health risks. Furthermore, measurement error is a limitation in environmental health studies [45]. Future studies could investigate the impact of measurement error on temperature-mortality relationship, including how this differs by exposure method. Existing studies showed different results where one study found Berkson-type error that reduced the logistic regression results less than 1% [46], whereas other studies stated that measurement error could result in biased estimates and contribute to uncertainty in the results [47, 48]. Also, future studies could evaluate different types of exposure methods according to the study region’s characteristics and data availability. A multi-city multi-country study suggested that the climate reanalysis dataset well represents the monitoring station temperature dataset, expect for the tropical regions where it showed a low performance [49], however that study did not include rural areas. A spatially refined exposure dataset was found to be more appropriate for locations far from the weather stations [11].

This study showed that using different temperature exposure methods can result in different heat-mortality risk. The heat-related mortality was higher when basing exposure on monitoring station data. These findings offer useful information to researchers, communities, and policy makers, on efforts to reduce the health burden from heat by highlighting the importance of exposure assessment methods in estimating risk and in comparing risks across populations (e.g., rural versus urban), which is important for the present day and estimates of risks under climate change.

## Supplementary Information

Supplementary materials
