# Supplementary material for: Heat-mortality relationship in North Carolina: Comparison using different exposure methods
Source: J Expo Sci Environ Epidemiol. 2023 Apr 7;33(4):637–45. doi: 10.1038/s41370-023-00544-y (PMC10403356; doi:10.1038/s41370-023-00544-y)
Supplement: Supplementary file 1 — Supplementary materials [file 41370_2023_544_MOESM1_ESM.docx]

**Supplementary Materials**

**
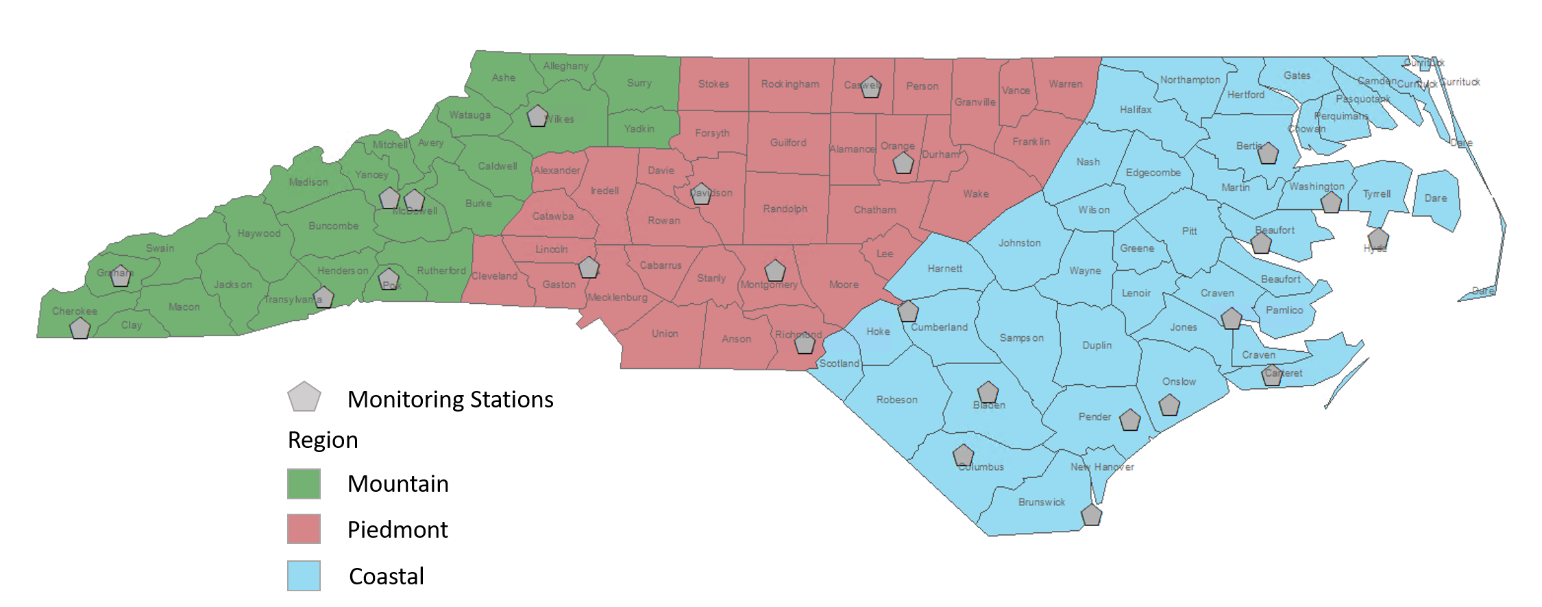
**

**Figure S1. Geographical location of 25 monitoring stations and different regions in North Carolina during 2000-2016.**

**
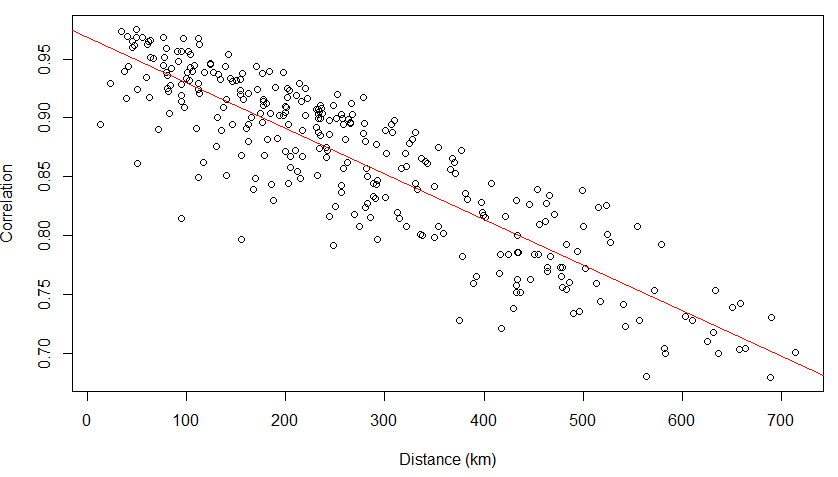
**

**Figure S2. Correlation and distance relationship for pairs of monitoring stations among total 25 monitoring stations.**


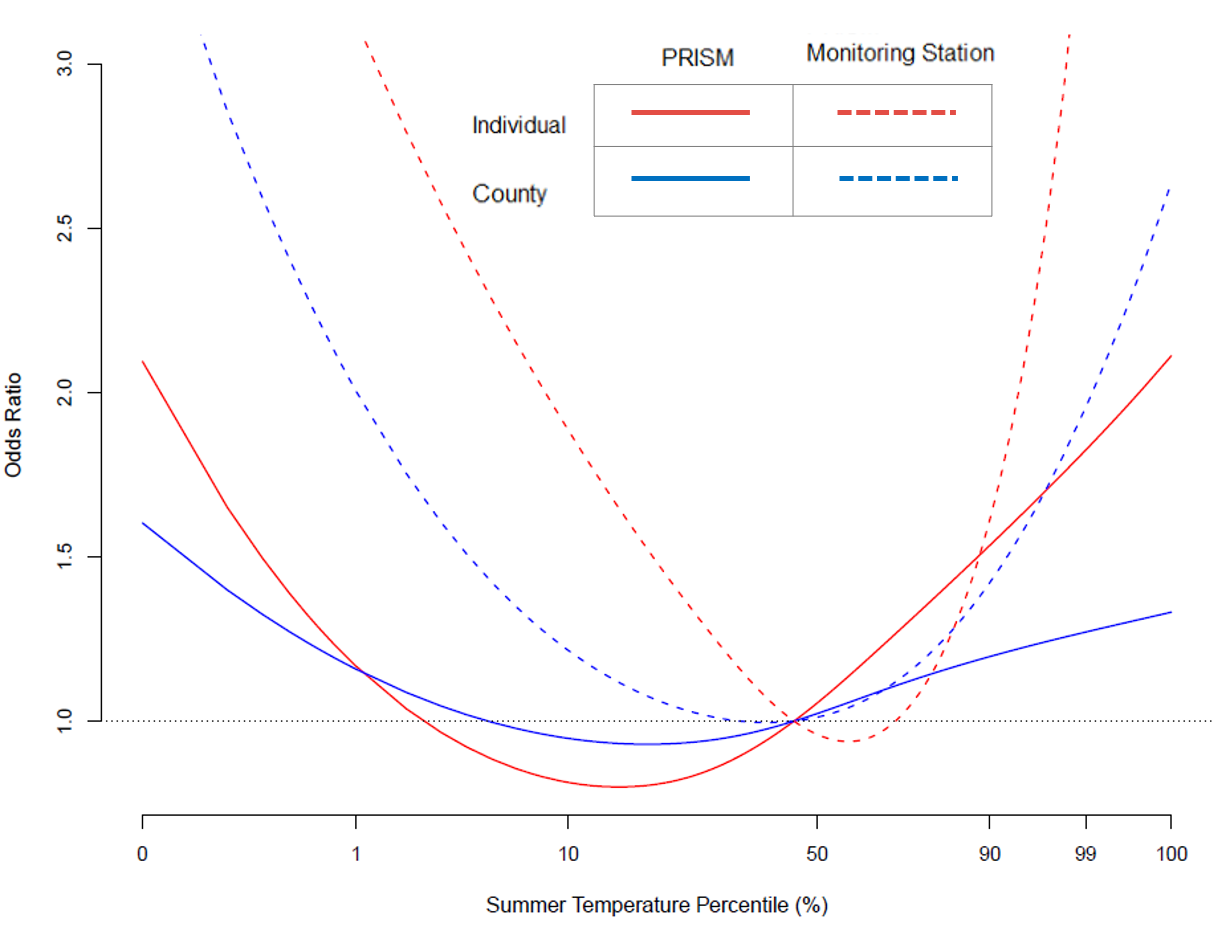
**Figure S3. Exposure-response curve for each exposure methods using all individuals.** Solid lines indicate results based the gridded, modeled temperature dataset (PRISM) and dashed lines reflect results based on monitoring station temperature. Blue lines indicate results based on exposure matched to individual residence, and red lines indicate results based on exposure matched to the residential county aggregated. The odds ratio was centered (OR=1) for the overall temperature mean value of 23.2°C.

**Table S1. Detailed summary statistics for estimated temperature exposure for mortalities included under different exposure methods**

|  |  | Temperature (°C) | | | | | | |
| --- | --- | --- | --- | --- | --- | --- | --- | --- |
|  |  | Mean | Median | Min | Max | 99^th^ | 90^th^ | 75^th^ |
| NC temperature | | 23.2 | 23.7 | 4.2 | 33.3 | 30.0 | 27.6 | 25.0 |
| PRISM | Individual residence | 23.4 | 23.9 | 5.1 | 33.0 | 29.8 | 27.9 | 26.2 |
|  | Residential county | 23.4 | 23.9 | 4.2 | 33.2 | 30.1 | 28.0 | 26.3 |
| Monitoring Stations | Individual residence | 23.6 | 23.8 | 4.4 | 35.0 | 30.0 | 28.3 | 26.7 |
|  | Residential county | 23.6 | 23.8 | 4.4 | 35.0 | 30.0 | 28.3 | 26.1 |

*Note:* The number of participants varied by exposure method (See Table 1).

**Table S2. Descriptive statistics for temperature data for different exposure methods using the subset of individuals with exposure assessments available under all methods (May–September, 2000–2016, n=109,569).**

|  |  |  |  |  |  |
| --- | --- | --- | --- | --- | --- |
|  |  | Individual residence (N=109,569) | | Residential county (N=118,848) | |
|  |  | PRISM (°C) | Monitoring Station (°C) | PRISM (°C) | Monitoring Station (°C) |
|  |  | Mean (SD) | Mean (SD) | Mean (SD) | Mean (SD) |
|  | Total | 23.4 (3.8) | 23.6 (3.9) | 23.6 (4.2) | 23.7 (4.0) |
| Region | Piedmont | 23.5 (3.6) | 23.6 (3.6) | 23.4 (3.6) | 23.6 (3.7) |
|  | Mountain | 20.7 (3.5) | 20.5 (3.4) | 20.0 (3.3) | 20.8 (3.5) |
|  | Coastal | 24.7 (3.5) | 25.2 (3.5) | 25.4 (3.9) | 25.4 (3.6) |
| Urbanicity | Urban Area | 24.3 (3.5) | 24.1 (3.6) | 24.5 (3.4) | 24.5 (3.5) |
|  | Urban Cluster | 23.6 (3.9) | 24.1 (3.8) | 24.1 (4.2) | 24.1 (3.9) |
|  | Rural | 22.9 (3.9) | 22.6 (4.0) | 22.3 (4.0) | 22.9 (4.0) |
| Race | Non-Hispanic White | 23.1 (3.8) | 23.0 (3.8) | 22.8 (3.9) | 23.2 (3.8) |
|  | Non-Hispanic Black | 24.4 (3.7) | 25.3 (3.7) | 25.5 (4.3) | 25.3 (3.9) |
|  | Hispanic | 23.7 (3.5) | 23.7 (3.4) | 23.6 (3.3) | 23.5 (3.4) |
|  | Non-Hispanic Asian/Hawaiian  Pacific Islander | 23.7 (3.7) | 24.3 (3.6) | 23.8 (3.6) | 24.1 (3.8) |
|  | Non-Hispanic Other | 24.3 (3.6) | 24.2 (3.9) | 24.2 (3.7) | 23.9 (3.7) |
| Sex | Female | 23.5 (4.0) | 23.9 (4.0) | 23.9 (4.4) | 24.0 (4.0) |
|  | Male | 23.3 (3.9) | 23.4 (3.9) | 23.2 (4.0) | 23.5 (3.8) |
|  |  |  |  |  |  |
| Age | < 65 years | 23.8 (3.9) | 24.4 (4.1) | 24.5 (4.6) | 24.4 (4.2) |
|  | ≥ 65 years | 23.2 (3.8) | 23.1 (3.7) | 23.0 (3.8) | 23.3 (3.8) |

*Note:* The number of participants varied by exposure method (See Table 1).

**Table S3. Heat related mortality (OR, 95% CI) estimated from conditional logistic regression models used within a case-crossover framework for different exposure methods using all individuals.**

|  | Odds Ratio (95% CI) | | | |
| --- | --- | --- | --- | --- |
|  | Temperature based on individual resident | | Temperature based on residential county | |
|  | PRISM | Monitoring Stations | PRISM | Monitoring Stations |
| Relative | 1.15 (1.14, 1.16) | 2.24 (2.22, 2.27) | 1.06 (1.05, 1.07) | 1.27 (1.25, 1.29) |
| Absolute | 1.23 (1.21, 1.26) | 2.21 (2.19, 2.25) | 1.09 (1.07, 1.11) | 1.43 (1.41, 1.48) |

*Note:* Odds ratio comparing the heat-mortality risk of relative temperature changes (99^th^ temperature percentile and 90^th^ temperature percentile) and absolute temperature changes (30.0°C and 27.6°C).

**Table S4. Heat related mortality (OR, 95% CI) by urbanicity estimated from conditional logistic regression models used within a case-crossover framework for different exposure methods using all individuals.**

|  | Odds Ratio (95% CI) | | | |
| --- | --- | --- | --- | --- |
|  | Temperature based on individual resident | | Temperature based on residential county | |
|  | PRISM | Monitoring Stations | PRISM | Monitoring Stations |
| Urban Area | 1.31 (1.25, 1.38) | 1.11 (1.02, 1.21) | 1.23 (1.16, 1.29) | 1.06 (1.00, 1.12) |
| Urban Cluster | 1.18 (1.12, 1.19) | 1.24 (1.14, 1.27) | 1.04 (1.00, 1.06) | 1.35 (1.27, 1.38) |
| Rural | 1.30 (1.24, 1.32) | 1.36 (1.26, 1.41) | 1.09 (1.03, 1.12) | 1.37 (1.29, 1.41) |

*Note:* Odds ratio comparing the heat-mortality risk of relative temperature changes (99^th^ temperature percentile and 90^th^ temperature percentile).
